# Athletic identity, anxiety, and depression in moderate to highly specialized female adolescent volleyball players

**DOI:** 10.3389/fpsyg.2025.1525074

**Published:** 2025-04-01

**Authors:** V. Claire Clark, Sophia M. Ulman, Ashley L. Erdman, Emily B. Gale, Joseph Janosky, Emily J. Stapleton

**Affiliations:** ^1^Scottish Rite for Children, Frisco, TX, United States; ^2^Department of Orthopaedic Surgery, University of Texas Southwestern Medical Center, Dallas, TX, United States; ^3^Department of Psychiatry, University of Texas Southwestern Medical Center, Dallas, TX, United States; ^4^Department of Athletic Training, Lasell University, School of Health Sciences, Newton, MA, United States

**Keywords:** mental health, specialization, athletic identity, anxiety, depression, female

## Abstract

**Introduction:**

Individuals strongly tied to their athletic sense of self, athletic identity, may have increased sport specialization and behaviors elevating injury risk, overtraining, and mental health concerns. No known studies have explored the relationship between athletic identity, specialization, and youth athletes’ mental health concerns, specifically anxiety and depression. This study assessed the relationship between athletic identity, specialization, and mental health symptoms among female, high school athletes.

**Methods:**

Athletic identity and mental health were assessed via the Athletic Identity Measurement Scale (AIMS) and Revised Children’s Anxiety and Depression Scale (RCADS). A sport participation survey recorded injury history, specialization, and training volume. Mann -Whitney U tests compared RCADS scores between high athletic identity (>54 total AIMS) and low athletic identity (<55 total AIMS; α = 0.05) athletes.

**Results:**

A total of 149 female volleyball athletes were included (16.0 ± 0.9 years), with 54.4% (81/149) classified as high athletic identity. Injury rates did not significantly differ between high and low athletic identity groups. Highly specialized athletes differed on multiple RCADS subscales, with high athletic identity correlated with greater separation anxiety (*p* = 0.012), generalized anxiety (*p* = 0.006), social phobia (*p* = 0.020), depression (*p* = 0.011), total anxiety (*p* = 0.005), and total anxiety and depression (*p* = 0.002). The moderately specialized group did not differ in RCADS scores between high and low athletic identity athletes.

**Discussion:**

Those with high athletic identity practiced more and had more anxiety and depressive symptoms than those with low athletic identity but were not at higher injury risk for injury. Providers should consider routine mental health screenings for high athletic identity athletes and promote psychoeducation on the importance of developing coping skills and diverse interests outside of one’s primary sport.

## 1 Introduction

Brewer et al. (1993) developed and popularized the concept of athletic identity, which has been defined as the degree of strength and exclusivity to which a person identifies with the athlete role and looks to others for confirmation of that role (Brewer and Cornelius, 2001, 2010; Brewer et al., 1993; Edison et al., 2021; Haraldsdottir and Watson, 2021; Lochbaum et al., 2022; Renton et al., 2021). Literature has also defined athletic identity as the extent to which an athlete devotes special attention to their sport relative to other engagements or activities in life (Edison et al., 2021). Therefore, athletic identity can be considered as the degree to which an individual identifies with and attends to their athletic role in comparison to other aspects of their life. All athletes are thought to embody an athletic identity, despite individual athlete and sport-specific differences (Renton et al., 2021). Additionally, athletic identity has been shown to increase through childhood, adolescence, and adulthood, and decrease when the individual’s competitive athletic career ends (Houle et al., 2010).

Brewer et al. (1993) theorized that strong athletic identity can be associated with both positive and negative factors in psychosocial development and functioning. When examining positive benefits, athletic identity can play an important social and cognitive role, which stems from individual emotional connection and feedback from teammates, coaches, parents, and spectators (Edison et al., 2021). As such, it may positively contribute to athletes’ emotional connection to their sport and can benefit their athletic performance outcomes, involvement in physical activity, commitment to training, level of enjoyment in sports, and sport goal orientation (Edison et al., 2021). Additionally, many athletes closely associate their athletic performance with their psychological health (Haraldsdottir and Watson, 2021). Specifically, Renton et al. (2021) found that stronger athletic identity is associated with positive health outcomes, longevity in sports, as well as improved self-esteem, confidence, and social relationships. Furthermore, athletic identity may also play a role in injury recovery, as it was positively associated with improved functional outcomes (Renton et al., 2021).

In contrast to the numerous aforementioned positive benefits, athletic identity has potentially negative implications as well. For instance, it may worsen the psychological impact of injury and increase competition-related stress and anxiety, which can directly influence athletes’ quality of life (Watson et al., 2022). Adolescent athletes’ own identities (Edison et al., 2021) and self-esteem (Haraldsdottir and Watson, 2021; Nippert and Smith, 2008) may be affected by a strong exclusivity in athletic identity because maintaining one’s athlete role may require the neglect of other identities, hobbies, and role responsibilities (Renton et al., 2021). The instability of individual identities is demonstrated by the finding that athletes with stronger athletic identity experience more emotional difficulty after retiring from or ceasing participation in sports (Brewer et al., 1993; Giannone et al., 2017). Giannone et al. (2017) found that athletes’ degree of athletic identity may be a risk factor for psychological disturbance after retiring from sport.

The negative impact of athletic identity after ceasing sport participation is particularly relevant in young athletes who sustain sports injuries as identity formation and exploration are pivotal developmental milestones during adolescence (Erikson, 1959; Manuel et al., 2002). Athletes with high athletic identity unilaterally identify with the sport role and are highly susceptible to identity foreclosure, or the tendency to avoid exploratory behaviors and cultivation of additional interests and social roles outside of sport due to one’s high commitment to the athlete role (Brewer and Petitpas, 2017). Athletic identity foreclosure is most pronounced in late adolescence and is responsive to changes in an athlete’s sport participation status (Brewer and Petitpas, 2017). Individuals with this unilateral identification to the athlete role are vulnerable to maladaptive behaviors and psychological distress, including increased substance use (Murray, 2001), burnout (Martin and Horn, 2013) and challenges with psychological adjustment to sport transitions (Baranoff et al., 2015; Brewer and Cornelius, 2010; Park et al., 2013; Ronkainen et al., 2016). Consequently, injuries may be detrimental to these athletes’ involvement in sport, self-esteem, and motivation due to significant impacts on one’s identity and lack of additional coping resources (Brewer and Cornelius, 2010; Petitpas and France, 2012).

Stronger athletic identity has been associated with depressive symptoms following sports-related injuries (Haraldsdottir and Watson, 2021; Manuel et al., 2002; Renton et al., 2021) because it disrupts the athlete role, provokes a loss of identity, and causes psychological distress (Brewer and Cornelius, 2010). Athletes with higher athletic identity may engage in rehabilitation overadherence, risking premature return to sport because they feel the need to perform behaviors and actions that are consistent with the athlete role, such as pushing through pain and not reporting pain (Tasiemski and Brewer, 2011; Weinberg et al., 2013). In adolescent athletic samples, females, injury severity, and athletic identity are associated with increased rates of depressive symptoms and negative emotional outcomes following injury (Manuel et al., 2002). Sex differences in the rates of depression following injury may be accounted for by the stress reactivity model, whereby females demonstrate increased vulnerability to stress-mediated emotional reactivity and depressive symptoms (Hammen, 2009). Additionally, athletes under 21 years of age with strong athletic identity have been found to experience more injury-related emotional trauma than those with weaker athletic identity (Padaki et al., 2018). Similar findings exist in studies of athletes following sports-related concussion (Collict et al., 2025; Dean, 2019). Athletes who experience persistent symptoms after sustaining a sports-related concussion may experience psychosocial consequences, including isolation and disturbances in mood and emotion (Dean, 2019). Collict et al. (2025) examined athletic identity disruption, a significant psychological consequence of sports-related concussion in athletes using the social identity model of identity change (SIMIC) as a theoretical lens to help understand the ways in which athletes navigate identity-related changes following sports-related concussion. SIMIC is a theoretical framework through which life events that precipitate identity-related changes can be understood (Haslam et al., 2019; Haslam et al., 2021). Collict et al. (2025) found that sports-related concussion disrupted athletes’ perceptions of their personal, social, and athletic identities, leading to psychosocial changes. They reported disruptions to their self-concept during recovery, such as an altered sense of personal identity, decreased confidence, and perceived changes to their role within social groups (Collict et al., 2025). Moreover, Tasiemski and Brewer (2011) examined the relationships between athletic identity, sport participation, and psychological adjustment in individuals with spinal cord injury. They concluded that social factors are important in these relationships, particularly in the link between sport participation and psychological adjustment. Similarly, Podlog et al. (2013) found that injured adolescent and collegiate athletes’ concerns about self-presentation and athletic identity predicted risky rehabilitation behaviors. Taken together, these studies underscore the potential deleterious effects of exclusive identification with the athlete role. Injury may also provide opportunities for personal growth (Brewer and Cornelius, 2010; Collict et al., 2025). Athletes may exhibit a protective mechanism of decreased athletic identity following severe injuries, so that they may maintain a positive self-image while they are unable to participate in sports (Brewer and Cornelius, 2010). Collict et al. (2025) found that some athletes in their study developed a revised self-concept which was independent of their ability to participate in sports. Those with more diverse and less sport-focused social identity and social networks were more successful in managing identity disruptions.

Despite the breadth of research demonstrating the benefits of delayed sport specialization for early sampling of activities and sports emphasizing fun, purposeful play activities, and overall development of physical skills (Cote and Hay, 2002; Wylleman and Lavallee, 2004), sport specialization, or the year-round intensive participation in a single sport at the exclusion of others (Watson et al., 2022), has recently surged in youth athletics, resulting in demands for increased competition and a more elite environment (Edison et al., 2021). Higher levels of early sport specialization have been associated with decreased quality of life, inadequate sleep, and increased daytime sleepiness (Watson et al., 2022). Moreover, Jayanthi et al. (2013) found that early sport specialization is linked to increased psychological stress, burnout, and earlier dropout from sport. Athletic identity, exclusivity, and negative affectivity tend to increase with an athlete’s specialization level, which may be a result of more specialized athletes quitting their non-primary sports (Choudhury et al., 2024). This recent emphasis on sport specialization is often attributed to the belief that elite sport achievement is attainable through intense sport-specific training and early specialization (Edison et al., 2021), but has not been shown to improve future athletic performance (Smucny et al., 2015; Walton et al., 2021). College scholarships, playing professional sports, and the desire for talent recognition are all factors which have likely led to increased sport specialization (Brenner et al., 2019; Brewer and Cornelius, 2001). Pressures to specialize may come from athletes, coaches, and parents (Walton et al., 2021), with coaching climates that emphasize winning, encourage immoral actions, and promote aggressive behaviors among athletes (Hoffmann et al., 2022).

Athletic identity increases with sport specialization, leading athletes to devote significant time to their sport and associate their self-esteem with their performance, which increase their risk for overuse injuries, burnout, perfectionism, and overtraining (Brewer and Cornelius, 2001; Edison et al., 2021; Haraldsdottir and Watson, 2021; Walton et al., 2021). Perfectionistic concerns, specifically, have been associated with burnout, psychological stress, poorer well-being, and higher anxiety in youth athletes (Hoffmann et al., 2022; Walton et al., 2021). The increase in training hours associated with sport specialization exposes athletes to social isolation, poor academic performance, less time with family, and greater anxiety (Brenner et al., 2019). Due to higher psychological, physical, and social demands on athletes, they may be at a higher risk for developing anxiety and depression (Weber et al., 2018; Wylleman et al., 2004).

Female athletes are especially at risk for developing mental health symptoms (Herrero et al., 2020; Rice et al., 2019; Weber et al., 2018; Yang et al., 2007), as they are more likely to specialize, participate in high competition volume, and be on a club team than male athletes (Post et al., 2017). To the best of our knowledge, rates of anxiety and depression and their relationship to athletic identity have not been specifically examined among elite female youth volleyball players. The present study was designed and executed in light of the particularly high and increasing incidence of female youth sport participation (Post et al., 2017), coupled with the significant associated risks for injury and psychosocial challenges in specialized athletes (Brenner et al., 2019; Brewer and Cornelius, 2010; Edison et al., 2021; Haraldsdottir and Watson, 2021; Hoffmann et al., 2022; Walton et al., 2021; Watson et al., 2022; Weber et al., 2018; Wylleman et al., 2004). Much of the literature on this subject is dated, and due to the growth in female sport specialization, this study aimed to assess the relationship between athletic identity, sport specialization, and mental health symptoms to expand upon psychological risk factors in specialized female athletes and provide more appropriate screening and targeted intervention. Additionally, while athletic identity, sport specialization, and mental health symptoms have all been investigated independently, the relationship between all three variables has not been studied. We hypothesized that this assessment would reveal that higher athletic identity and increased sport specialization would lead to increased anxiety and depression symptoms in these athletes.

## 2 Materials and methods

This cross-sectional study was conducted with approval from the local Institutional Review Board (IRB# 082010–134). Participants reporting musculoskeletal injury within the previous 3 months or a diagnosis of orthopaedic conditions that would limit their ability to perform the required tasks were excluded. Participation in this study was voluntary, and all participants provided informed assent and/or consent prior to the initiation of study procedures. Additionally, contact information for the primary investigator was provided to all participants prior to survey initiation.

### 2.1 Procedures

The administered surveys were chosen to assess specialized adolescent female volleyball players’ current psychosocial and sport functioning. Participants were consented onsite prior to data collection at two large, invitation-only national volleyball tournaments that took place in December of 2021 and December of 2022. Participants provided informed consent electronically on their personal device and were directed immediately to the surveys upon completion of the consent form and verification by a study team member. If the participant did not have a personal device, a tablet was provided. Participants were tested on the first day of each of the two events. The first day consisted of workshops, skills stations, movement screens, and vendor demonstrations. Data collection for this study was integrated into a series of stations the athletes moved through with their parents, as interested. Competition began on the following day. All participants were in grades 9 through 12 and between the ages of 13 and 18 years. Data was collected and stored using REDCap, a secure, web-based electronic data capture tool at Scottish Rite for Children (Research Electronic Data Capture; Vanderbilt University). A convenience sample of 149 female volleyball athletes were included in this study. Participants’ demographic, sport participation, and injury history characteristics are included in Table 1.

**Table 1:** Demographic, sport participation, and injury history measures collected from the sport participation survey.

Variable	*N* (%)
Demographics	
Age (years) (Mean ± SD)	16.0 (± 0.9)
Range (years)	13.6–17.9
Grade level (*n* = 149)	
9th	21 (14.1)
10th	45 (30.2)
11th	52 (34.9)
12th	31 (20.8)
Race (*n* = 119)	
White	98 (82.4)
Black	2 (1.7)
Asian	3 (2.5)
White/Hispanic	4 (3.4)
Mixed race	12 (10.1)
Sport participation	
Years in sport (Mean ± SD)	6.6 (± 2.1)
Months active per year (Mean ± SD)	10.8 (± 1.4)
Take an offseason (*n* = 134)	44 (29.5)
Number of practices per week (Mean ± SD)	3.9 (± 1.6)
HSS Pedi-FABS* Score (Mean ± SD)	23.8 (± 4.3)
Competition level (*n* = 149)	
School	6 (4.0)
Club / select / travel team	121 (81.2)
National elite	22 (14.8)
Specialization level (*n* = 133)	
Low	0 (0)
Moderate	42 (28.2)
High	91 (61.1)
Multi-sport participation	42 (28.2)
Injury history	
Past knee injury	32 (21.5)
Past concussion	12 (8.1)
Recent injury	59 (39.6)

* HSS Pedi-FABS = The Hospital For Special Surgery Pediatric Functional Activity Brief Scale.

### 2.2 Measures

Participants were asked to complete self-report questionnaires, including a demographic, sport participation, and injury history questionnaire, the Hospital for Special Surgery Pediatric Functional Activity Brief Scale (HSS Pedi-FABS) (Fabricant et al., 2013; Wagner et al., 2019), the Athletic Identity Measurement Scale (AIMS) (Brewer and Cornelius, 2001; Brewer et al., 1993), and the Revised Children’s Anxiety and Depression Scale (RCADS) (Chorpita et al., 1998). The HSS Pedi-FABS questionnaire is an 8-item, generalizable measure of activity in children ages 10–18 (Fabricant et al., 2013; Wagner et al., 2019).

Demographic, sport participation and injury history items included: age, grade, years in sport, training volume (years in sport, days per week, weeks per year, and weeks off per year), competition level (school, club/select/travel team, national elite), single- or multi-sport participation, and injury history (past knee injury, past concussion, and if they experienced an injury in the last year). Sport specialization was assessed through a three-question survey used by Jayanthi et al. (2015). Athletes’ degree of sport specialization was categorized as low, moderate, or high based on the participants’ answers to 3 survey questions: “Can you pick a main sport?,” “Did you quit other sports to focus on a main sport?,” and “Do you train >8 months in a year?” To each question, participants could answer either “yes” or “no.” The degree of specialization was computed as the sum of the 3 questions (Jayanthi et al., 2015).

Athletic identity was measured via the AIMS. The AIMS is validated to measure athletic identity (Brewer and Cornelius, 2001; Brewer et al., 1993) in elite athletes, recreational athletes, and non-athlete populations, and has been demonstrated to have robust internal consistency and test–retest reliability using youth student samples (Brewer et al., 1993; Edison et al., 2021). The psychometric integrity of the AIMS has been well supported in the literature, with evidence of test–retest reliability (*r* = 0.89 over a two-week period) and internal consistency (Chronbach’s alpha = 0.81 to 0.93) (Brewer and Cornelius, 2001; Brewer et al., 1993; Good et al., 1993; Mitchell et al., 2021). It captures the athlete’s relationship to their primary sport by having the athlete rate 10 statements on a 7-point Likert scale, with possible responses ranging from ‘strongly disagree’ to ‘strongly agree.’ The total AIMS score was computed as the sum of values from all 10 statements in the questionnaire. The AIMS was further divided into subfactors: Social Identity, Exclusivity, and Negative Affectivity. Participants who have higher achievement standards and are the most invested in athletics relate the most with the AIMS statements (Lochbaum et al., 2022).

The 47-item RCADS questionnaire was used to assess mental health symptomology. The RCADS evaluates childhood (ages 8 to 18) anxiety disorders and depression by assessing the following subscales: separation anxiety, social phobia, generalized anxiety disorder, panic disorder, obsessive compulsive disorder, and major depressive disorder (Chorpita et al., 1998). The RCADS has demonstrated the ability to successfully identify children and adolescents with anxiety and depressive disorders in clinical and non-clinical populations (Chorpita et al., 1998; Chorpita et al., 2005), and has demonstrated robust internal consistency (Piqueras et al., 2017). The RCADS was selected as the measurement tool for mental health symptoms to reduce responder burden, as the tool assesses both anxiety and depressive symptoms in a single scale. It is also a good tool for diverse community-based settings as it is a free resource and has been translated into 30 languages (e.g., Dutch, Greek, Korean, Spanish, Swedish, and Turkish) (Revised Child Anxiety and Depression Scale, n.d.).

### 2.3 Statistical analysis

The mean total AIMS score (54) was used as a cutoff to divide participants into high and low AIMS score groups, in accordance with recently published studies, given there is no established threshold (Choudhury et al., 2024; McGinley et al., 2022; McGinley J. J. et al., 2024; McGinley J. et al., 2024). Therefore, total AIMS >54 of 70 was considered a “high” AIMS score. Data were analyzed for normality via the Shapiro–Wilk test, which was significant. Thus, Mann–Whitney U tests were performed to identify significant differences in demographics, sport participation, specialization level, and RCADS scores between the groups, and *p*-values were used to report significance (*α* = 0.05). Statistical analysis was conducted using R Programming (version 4.3.0, R Development Core Team, www.r-project.org) and SPSS (version 24).

## 3 Results

There were no academic grade-related differences in sport participation (practices per week, *p* = 0.058; months played per year, *p* = 0.342). Grade-related differences were also absent in HSS Pedi-FABS (*p* = 0.138) and AIMS (*p* = 0.195) scores, as well as with all RCADS subscores, the Total Anxiety Score (TAS, *p* = 0.325), and the Total Anxiety and Depression Score (TAD, *p* = 0.371). However, differences based on sport participation characteristics were identified. Specifically, participants who quit other sports to focus on their primary sport had a higher total AIMS score (55.6 ± 9.5 vs. 51.0 ± 10.5; U = 1388.0; *p* = 0.002), higher exclusivity (19.2 ± 5.0 vs. 16.8 ± 4.9; U = 1445.0; *p* = 0.005), and higher negative affectivity (10.9 ± 2.6 vs. 9.5 ± 3.0; U = 1481.0; *p* = 0.008) than those who did not quit other sports. Additionally, multi-sport athletes had higher social identity scores than single-sport athletes (20.0 ± 2.6 vs. 19.1 ± 3.6; U = 1377.5; *p* = 0.013) and demonstrated higher separation anxiety than single-sport athletes (48.7 ± 10.4 vs. 44.3 ± 7.5; U = 1393.5; *p* = 0.022).

### 3.1 Athletic identity

The mean AIMS score was 54.4, with a range of 15 to 70. For analyses between AIMS groups, 54.4% (81/149) of athletes were considered to have high athletic identity (Total AIMS score > 54 of 70). Participants in the high and low athletic identity groups did not significantly differ in the number of practices per week (4.1 ± 1.9 vs. 3.6 ± 1.2 practices; *p* = 0.214). Alternatively, significant differences in RCADS scores were found and are reported in Table 2. High athletic identity athletes reported increased Generalized Anxiety, Social Phobia, Major Depression, Total Anxiety, and Total Anxiety and Depression. The high and low athletic identity groups did not significantly differ in reporting a knee injury in the past (*p* = 0.841) or a history of any injury within the past year (*p* = 0.734).

**Table 2:** Comparative analysis of anxiety and depression scores, assessed by the Revised Children’s Anxiety and Depression Scale (RCADS), between participants in the high and low athletic identity groups.

	High athletic identity***		Low athletic identity***			
Variable	Mean	SD	Mean	SD	U	*p*-value
Separation anxiety	46.51	8.74	44.51	8.26	2288.5	0.073
Generalized anxiety	42.81	9.68	38.66	7.61	2067.0	0.009**
Panic disorder	46.98	9.75	45.00	9.18	2422.5	0.202
Social phobia	47.62	11.01	43.31	10.65	2153.5	0.022*
Obsessive-compulsive disorder	42.58	9.54	40.19	7.43	2432.5	0.217
Major depressive disorder	43.64	10.39	38.26	9.27	1870.5	0.001**
Total anxiety	44.31	10.65	40.25	9.22	2122.0	0.016*
Total anxiety and depression	43.91	10.96	39.13	9.47	2002.5	0.004**

* Significance at α = 0.05. ** Significance at α = 0.01. ***High Athletic Identity = > 54 total AIMS; Low Athletic Identity = <55 total AIMS. Bolded values indicate significance at α = 0.05.

### 3.2 Mental health differences by specialization status

Of the 133 participants who indicated a level of sport specialization, 31.6% (42/133) of the athletes were identified as moderately specialized and 68.4% (91/133) were identified as highly specialized. No participants (0.0%; 0/133) were identified for the low specialization group. Thus, for group comparisons, the moderate specialization group was compared to the high specialization group. The high specialization group reported significantly more practices per week (4.03 ± 1.72 vs. 3.36 ± 1.28 practices; *p* = 0.046), as well as higher total AIMS scores (55.33 ± 9.55 vs. 50.88 ± 10.62; *p* = 0.004), exclusivity (18.99 ± 5.07 vs. 16.81 ± 4.78; *p* = 0.011), and negative affectivity (10.80 ± 2.68 vs. 9.43 ± 2.93; *p* = 0.008). The two groups did not significantly differ in RCADS total score (*p* = 0.590), any RCADS subscores, a history of a knee injury in the past (*p* = 0.512), or a history of any injury within the past year (*p* = 0.852).

**Figure 1:**
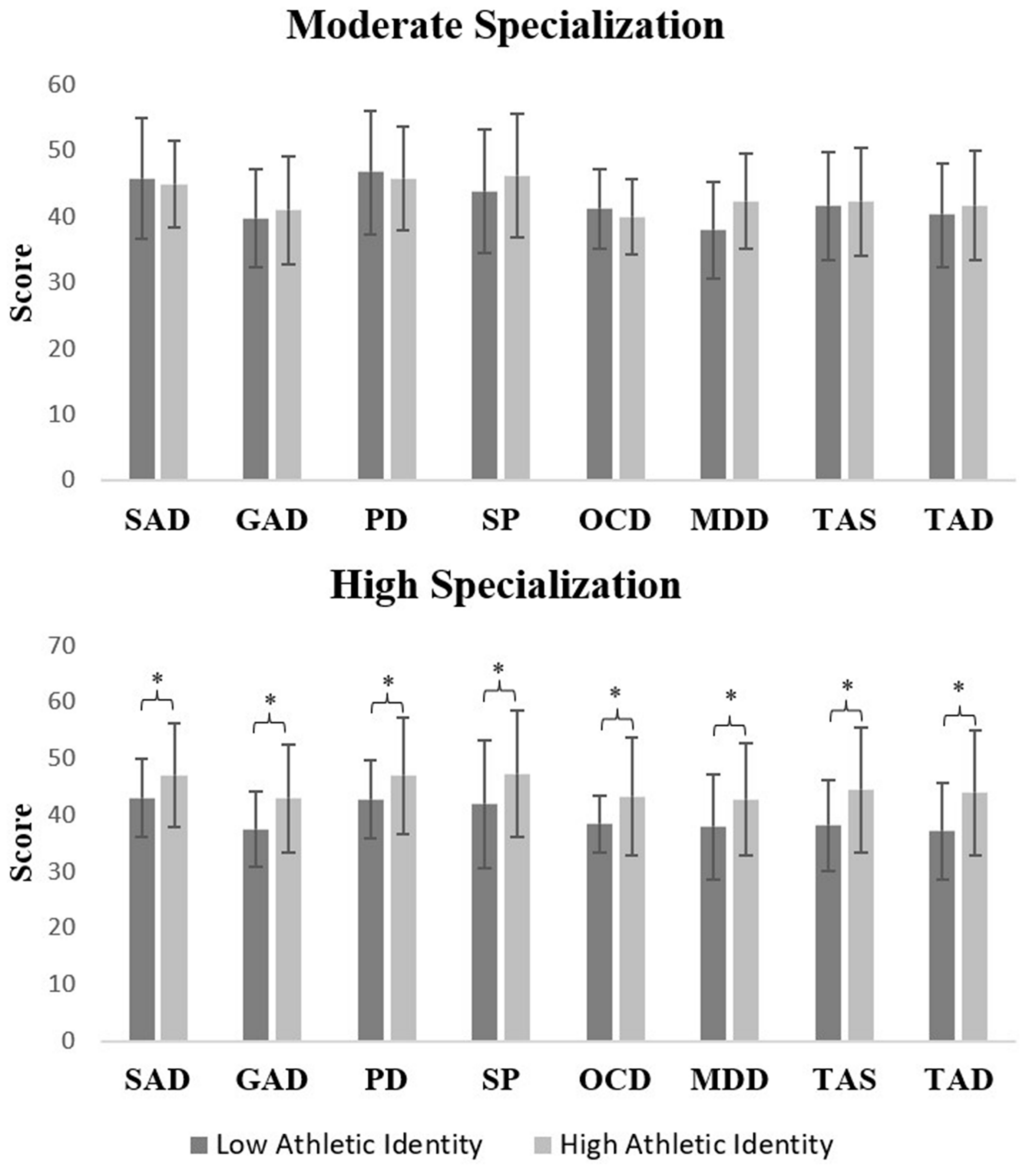
Differences in Revised Children’s Anxiety and Depression Scale (RCADS) scores by athletic identity group for moderate and highly specialized athletes. Significant athletic identity group differences noted with bracket and associated *p*-value. RCADS scores include: SAD, Separation Anxiety; GAD, Generalized Anxiety; PD, Panic Disorder; SP, Social Phobia; OCD, Obsessive-Compulsive Disorder; MDD, Major Depressive Disorder; TAS, Total Anxiety Score; and TAD, Total Anxiety and Depression Score. * Significance at α = 0.05. ** Significance at α = 0.01.

Within the moderate specialization group, those with high athletic identity (35.7%; 15/42) were compared to those with low athletic identity (64.3%; 27/42) and differed only in activity level (HSS Pedi-FABS score 25.7 ± 3.0 vs. 22.5 ± 5.2; *p* = 0.042). There were no other significant differences between moderately specialized athletes in the high versus low athletic identity groups. Similarly, within the high specialization group, those with high athletic identity (60.4%; 55/91) were compared to those with low athletic identity (39.6%; 36/91) and differed across multiple RCADS subscales: Separation Anxiety (47.1 ± 9.2 vs. 43.0 ± 7.0), Generalized Anxiety (43.0 ± 9.6 vs. 37.4 ± 6.7), Panic Disorder (47.1 ± 10.3 vs. 42.7 ± 6.9), Social Phobia (47.3 ± 11.2 vs. 41.9 ± 11.3), Obsessive Compulsive (43.2 ± 10.5 vs. 38.4 ± 5.0), Major Depression (42.8 ± 9.9 vs. 37.9 ± 9.3), Total Anxiety (44.5 ± 11.0 vs. 38.2 ± 8.1), and Total Anxiety and Depression (43.9 ± 11.1 vs. 37.2 ± 8.5). See athletic identity group differences in RCADS scores by specialization group in Figure 1 below. Lastly, within the high specialization group, the high and low athletic identity participants did not differ in age (years, *p* = 0.527), experience (years in sport, *p* = 0.825), training volume (months played out of the year, *p* = 0.444; and practices per week, *p* = 0.715), or activity level (HSS Pedi-FABS score, *p* = 0.907).

### 3.3 Injury history

141 participants reported an injury history, with 41.8% (59/141) reporting that they sustained an injury within the past year. Among participants who had sustained an injury within the past year, AIMS and RCADS scores did not significantly differ from those who had not sustained an injury. There were no significant differences in specialization status (high or moderately specialized athletes; *p* = 0.852) or AIMS level (high or low AIMS group; *p* = 0.734) in those who had sustained an injury in the past year.

Additionally, 22.7% (32/141) of participants reported that they sustained a knee injury at some point in the past. These participants’ AIMS and RCADS scores did not significantly differ from those of the participants who had not sustained a knee injury. However, participants who reported at least one knee injury in the past were older (16.46 ± 0.78 vs. 15.93 ± 0.84 years; *p* = 0.001). There were no significant differences in specialization status (*p* = 0.512) or AIMS level (*p* = 0.841) in those who reported knee injuries.

## 4 Discussion

The purpose of the current study was to assess the relationship between athletic identity, sport specialization, and mental health symptoms, and expand upon the psychological risk factors among specialized, high school, female volleyball athletes. We found that highly specialized athletes practiced more per week and had higher total athletic identity, exclusivity, and negative affectivity than moderately specialized athletes. However, highly and moderately specialized athletes did not significantly differ in anxiety or depression scores. The present study identifies a positive relationship between athletic identity and increased mental health concerns. We found that, when comparing high athletic identity and low athletic identity athletes, high athletic identity participants practiced more per week and had more anxiety and depressive symptoms as measured by the RCADS. We did not, however, find a relationship between high athletic identity injury history, in contrast to findings from Bell et al. (2018). Our results noted increased mental health symptoms with increased sport specialization and athletic identity. Within highly specialized athletes, participants with high athletic identity scored significantly higher across multiple subscales of the RCADS than participants with low athletic identity (Figure 1).

Our results are consistent with identity development across adolescence, as athletic identity is thought to change with age, increasing through adolescence, plateauing during young adulthood, and decreasing when an athlete no longer plays sports (Houle et al., 2010). This is commonly attributed to social identity theory, which holds that identity recognition depends on one’s position within their social environment, and can shift throughout their life and career (Stets and Burke, 2000). Previous research has hypothesized that athletic identity increases with sport specialization (Brewer and Cornelius, 2001; Edison et al., 2021; Haraldsdottir and Watson, 2021; Walton et al., 2021; Choudhury et al., 2024). The present study supports this belief, noting a significantly higher average athletic identity in the high specialization group compared to the moderate specialization group, as more specialized athletes have an exclusive focus on one sport and their identity becomes centered around that specific role.

With high-level sport participation becoming increasingly more common (Edison et al., 2021; Haraldsdottir and Watson, 2021), the potential impact of specialization and athletic identity on athletes’ mental health continues to become more relevant. We believe high athletic identity, rather than specialization, is the larger contributor to mental health concerns. The association between high athletic identity and mental health difficulties has been well-documented (Appaneal et al., 2009; Brewer and Cornelius, 2010; Cox et al., 2017; Haraldsdottir and Watson, 2021; Mainwaring et al., 2010; Manuel et al., 2002; Renton et al., 2021; Smith et al., 1990). Based on previous literature and our findings, we believe that high athletic identity, rather than high specialization, is the main contributor to mental health symptoms. When comparing the high athletic identity group to the low athletic identity group, we found significant differences on multiple RCADS subscales, indicating that athletic identity impacts mental health symptoms.

In contrast, when we compared the high specialization group to the moderate specialization group, we did not find any differences in mental health scores. Previous research has found that specialization increases risk for psychosocial concerns, including anxiety, as well as inadequate sleep, social isolation, poor academic performance, and decreased quality time with family, likely due to the training demands of specialization (Brenner et al., 2019). Additionally, Watson et al. (2022) found decreased quality of life, increased daytime sleepiness, and lower sleep quality among specialized athletes and they hypothesized that these differences were related to increased injury rates connected with specialization. Of note, Watson et al. (2022) did not specifically evaluate anxiety, depression, or levels of athletic identity. In the present study, we did not find a significant difference in rates of previous injury between the high athletic identity and low athletic identity groups, whereas we noted significant differences in RCADS scores in the high and low athletic identity groups without the presence of different rates of injury. Therefore, while specialization may impact psychosocial and mental health concerns in these athletes, our findings suggest that high athletic identity, rather than injury history tied to specialization, contributes to mental health difficulties.

While we found increased mental health concerns among highly specialized athletes in our study, the athletes were not at diagnostic or clinical levels of mental health disorders assessed by the RCADS. Our study supports the general understanding that sport participation has benefits to mental health. Additionally, our comparison of the moderate and high specialization groups resulted in no differences between high and low athletic identity athletes in the moderate specialization group. In the high specialization group, we noted significantly lower mental health symptoms in the low athletic identity group compared to the high athletic identity group. This indicates that having low athletic identity may be protective against mental health concerns. This may be due to these athletes generally having a more diverse set of interests and sense of self outside of sports, compared to high athletic identity athletes, buffering against the potential negative consequences of the high training volume as well as the increased risk for social isolation, poor sleep, and decreased quality time with family (Brenner et al., 2019; Watson et al., 2022).

This study has several limitations to consider. First, this is a cross-sectional study that collected a convenience sample at a single timepoint for each athlete and relied on self-reported data from athletes of a single sport. While a power analysis was not conducted *a priori,* achieved power was computed to be 0.78 across the full sample and 0.86 across the high specialization group for the primary investigation of whether differences exist in anxiety and depression scores by high or low AI. Future work should similarly investigate a larger, more diverse population, especially including athletes of low or moderate specialization status. Additionally, data was collected at a high-level national volleyball tournament, where participants’ anxiety may have been increased by various pressures, including the desire to perform well, be noticed by college recruiters, and other anxiety-provoking factors. Future study design allowing for prospective data collection and multiple follow up opportunities throughout the course of athletes’ high school careers and away from competitive environments may help to offset the challenges of these limitations. Furthermore, there was also no established cutoff for “High” and “Low” AIMS scores in the literature, so a cutoff value was chosen based on the sample mean. Establishment of population cut-offs in the literature would benefit future research using AIMS to quantify athletic identity. Finally, causality cannot be established for the relationships in this study.

Providers should consider routine mental health screenings for high athletic identity youth athletes, especially females. Increased psychoeducation on the importance of developing coping skills and diverse interests outside of one’s primary sport are also advised. Late adolescence is an important period of identity formation, so it is important to encourage adolescents to delay specialization, to prevent the negative psychosocial consequences of high athletic identity when they experience sport cessation or injuries. Petitpas and France (2010) provided several recommendations for practitioners working with young athletes in identity foreclosure. They suggested that practitioners understand the role sport participation plays in young athletes’ identities, do not directly challenge the efficacy of an exclusive commitment to sport roles, help athletes move from extrinsic to intrinsic motivation, work with coaches and administrators to understand and plan educational strategies, and establish strong counseling relationships with athletes and assist them through a process of self-disclosure, feedback, and awareness (Petitpas and France, 2010). Other possible interventions include Rational Emotive Behavior Therapy (REBT) (Knapp et al., 2023) and Cognitive Behavioral Therapy (CBT), as both of these evidence based treatments have been found to be successful in supporting the psychological health in adolescent athletes (McCarthy, 2018; Wood et al., 2018). Future research is warranted to expand upon the current understanding of the relationship between specialization, athletic identity, and mental health concerns in youth athletes. Qualitative research may be beneficial to expand the current understanding of athletic identity. Moreover, exploring the relationship between the constructs of passion, as measured by the Passion Scale (Vallerand et al., 2003) and motivation on athletic identity may help further expand what particular factors put young athletes at risk for the development of a more rigid sense of self and the accompanying challenges to psychological wellbeing. Furthermore, future investigations into psychobehavioral interventions, such as REBT and CBT, on the outcomes of athletes at an increased risk for psychological challenges due to high athletic identity may be beneficial.

## Data Availability

The raw data supporting the conclusions of this article will be made available by the authors, without undue reservation.
